# Do equestrian helmets prevent concussion? A retrospective analysis of head injuries and helmet damage from real-world equestrian accidents

**DOI:** 10.1186/s40798-019-0193-0

**Published:** 2019-05-24

**Authors:** Thomas A. Connor, J. Michio Clark, Jayaratnam Jayamohan, Matt Stewart, Adrian McGoldrick, Claire Williams, Barry M. Seemungal, Rebecca Smith, Roy Burek, Michael D. Gilchrist

**Affiliations:** 1 School of Mechanical & Materials Engineering, University College Dublin, Belfield, Dublin 4 Ireland; 2 Charles Owen & Co, Wrexham, UK; 3 Department of Neurosurgery, John Radcliffe Hospital, Oxford, UK; 4 Irish Horseracing Regulatory Board, The Curragh, Co. Kildare Ireland; 5 British Equestrian Trade Association, Wetherby, UK; 6 Brain and Vestibular Group, Charing Cross Hospital Campus, Imperial College London, London, UK

**Keywords:** Concussion, Equestrian, Riding, Helmet, Head injury, Certification standards

## Abstract

**Objectives:**

To collect and analyse helmets from real-world equestrian accidents. To record reported head injuries associated with those accidents. To compare damage to helmets certified to different standards and the injuries associated with them.

**Methods:**

Two hundred sixteen equestrian helmets were collected in total. One hundred seventy-six helmets from amateur jockeys were collected via accident helmet return schemes in the UK and USA, while 40 helmets from professional jockeys were collected by The Irish Turf Club. All helmet damage was measured, and associated head injury was recorded.

**Results:**

Eighty-eight percent (189) of equestrian fall accidents returned an injury report of which 70% (139) reported a head injury. Fifty-four percent (75) of head injury cases had associated helmet damage while 46% had no helmet damage. Reported head injuries consisted of 91% (126) concussion, 4% (6) skull fractures, 1 (0.7%) subdural hematoma, 1 (0.7%) cerebral edema and 5 (3.6%) diffuse axonal injury (DAI). It is also shown that helmets certified to the most severe standard are overrepresented in this undamaged group (*p* <0.001).

**Conclusions:**

It is clear that despite jockeys wearing a helmet, large proportions of concussion injuries still occur in the event of a jockey sustaining a fall. However, the data suggest it is likely that helmets reduce the severity of head injury as the occurrence of skull fracture is low. The proportion of undamaged helmets with an associated head injury suggests that many helmets may be too stiff relative to the surface they are impacting to reduce the risk of traumatic brain injury (TBI). It may be possible to improve helmet designs and certification tests to reduce the risk of head injury in low-severity impacts.

## Key Points

- Seventy percent of all reported equestrian fall accidents resulted in a head injury, of which 91% were concussions.
- Helmets certified to more stringent certification tests were more likely to be undamaged in head injury cases, suggesting they may be too stiff to prevent concussion head injury.
- Helmet designs and certification tests may need to be improved to reduce the risk of head injury in low-severity impacts.

## Background

The primary purpose of equestrian helmets is to reduce the risk of head injury to a rider during an impact by attenuating the impact energy. Helmet designs are a product of standard tests by which they are certified, as well as the styles that people are prepared to wear. They must perform in a range of environments (wet, cold and hot) and be comfortable to wear.

Equestrian sports are high risk [1–7]. Indeed, the majority of professional jockey fatalities result from head injury where reported rates of concussion or mild traumatic brain injury (mTBI) are higher than those in boxing and American football [8]. Traumatic brain injury (TBI) is one of the main causes of death in adults under the age of 45, and survivors of such injuries can suffer long-term neurological disability, which has significant public health and societal implications [9–11].

Helmet certification tests have changed little over the last three decades. Pass criteria are designed to protect against skull fracture based primarily on primate studies [12, 13], without due regard for TBI and concussion injuries. However, since then, our understanding of impact biomechanics has advanced greatly [14]. Additionally, there is a growing public awareness of TBI and the risk of exposure in sport. Nevertheless, there is still a dearth of data regarding real-world equestrian accidents, helmet performance, helmet certification tests and how these correlate with head injury.

The aim of this retrospective study has been to collect and analyse equestrian helmets involved in real-world accidents and determine if any relationship existed between measured helmet damage and any reported head injury. Helmets certified to different standards were compared to see how reported injuries related to measured damage.

## Methods

### Sample

A total of 216 helmets were collected via damaged helmet return schemes in the UK and USA. The damaged helmet return schemes were run by the British Equestrian Trade Association (BETA) and Charles Owen. Essentially, helmet users were encouraged to exchange damaged helmets following an accident in return for a discount on a new helmet. The Irish Turf Club collected helmets from professional jockeys involved in serious race accidents. Helmets were collected between September 2015 and June 2018. Thirty different helmet models manufactured by Charles Owen, Champion, Gatehouse, Park Gate, Harry Hall, Kep, LAS and Pferde Sport were analysed. Helmets were manufactured between February 2002 and January 2017.

### Accident reports

One hundred eighty-nine individual accident report forms were provided, by either the riders or their doctors. However, these were not standardised forms and the level of recorded detail was different in each case. Where reported, all associated injuries, including skull fracture, concussion, facial and soft tissue injuries, were recorded. The criteria used to diagnose concussion was not reported and so the severity of this injury is not known. The type of equestrian activity and impact surface condition at the time of the accident was also recorded. All accident reports used in this study were collected retrospectively and were not designed with this study in mind.

### Helmet construction and certification tests

All 216 helmets that were examined had been certified to one or more of four standards by the British Standards Institute (BSI), the Snell Memorial Foundation, the American Society for Testing and Materials (ASTM) or by Publicly Available Specification (PAS). Current standards for equestrian helmets are BS EN1384: 2017 [15], Snell E2016, PAS015:2016 [16] and ASTM F1163–15 [17]. Many helmets in this study were certified to earlier versions of these standards, although only small changes to impact velocities and pass/fail criteria were made to tests over the 15-year period in which these helmets were manufactured. Table 1 gives a summary of the impact conditions and pass/fail criteria for each current standard.

**Table 1:** Impact surfaces and pass criteria for the four equestrian helmet standard tests (*g* = 9.81 m/s^2^)

Standard	Anvil	Impact velocity (m/s)	Pass criterion (g)	Total no. of impact locations (all anvils)
BS EN1384: 2017	Flat	5.94	< 250	3
	Hemi	–		
	Hazard	–		
Snell E2016	Flat	6.26	< 275	4
	Hemi	5.60		
	Hazard	5.24		
PAS 015: 2016	Flat	5.94	< 250 and < 225 Average	4
	Hemi	–		
	Hazard	5.40		
ASTM F1163-15	Flat	5.94	< 300	4
	Hemi	–		
	Hazard	5.40		

**Fig. 1:**
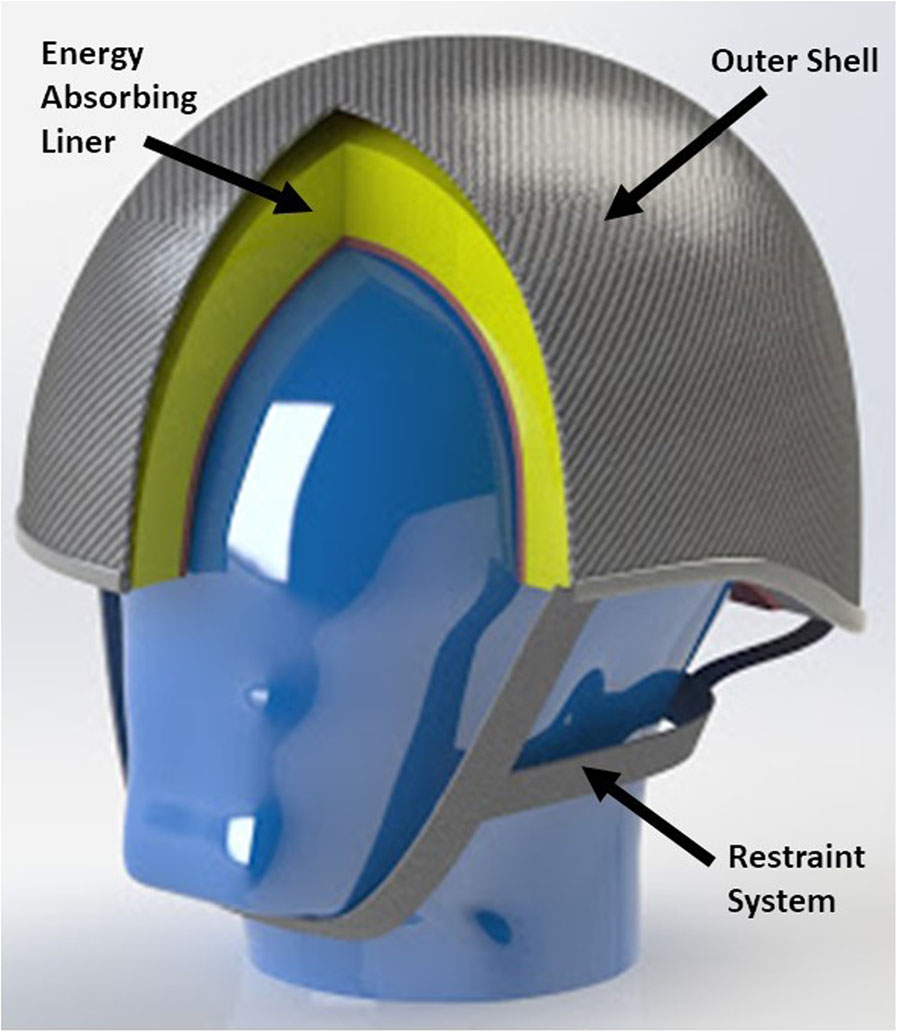
Main components of an equestrian helmet

Equestrian helmets have a tough outer shell, an energy absorbing liner, comfort padding and a restraint system that keeps the helmet in place. Helmets are composed of an outer shell made from plastic, usually acrylonitrile butadiene styrene (ABS) or a fibre reinforced plastic composite, and an energy absorbing liner usually made from expanded polystyrene (EPS) or expanded polypropylene (EPP). The energy absorbing liner is affixed to the outer shell (see Fig. 1).

**Fig. 2:**
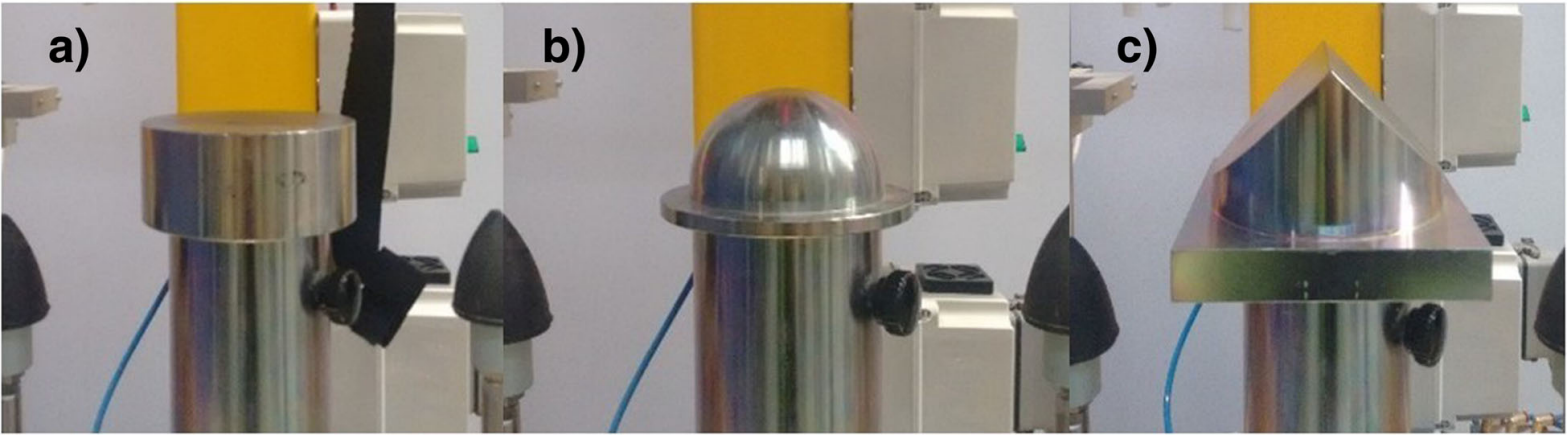
**a** Standard steel flat anvil. **b** Standard steel hemispherical anvil. **c** Standard steel hazard anvil

Helmets are widely used by professional and recreational horse riders alike. Indeed, in many cases, it is mandatory for a competing jockey to wear a helmet that is certified to a particular standard. The primary function of equestrian helmet standard tests is to evaluate performance against impact. They ensure that a helmet meets a minimum level of performance, although the tests do not necessarily recreate specific accident scenarios. Helmet performance characteristics are evaluated by creating simplified impacts in the laboratory (i.e., simpler than real-world impacts) and involve a helmeted human surrogate headform being dropped onto a rigid steel surface (anvil). All current standard tests commonly use a flat steel anvil impact surface (Fig. 2a), but some specify additional impacts onto hemispherical (Fig. 2b) and hazard anvils (Fig. 2c). To pass the test, a headform must not exceed a specified peak acceleration (peak g).

### Visual inspection versus CT imaging

**Fig. 3:**
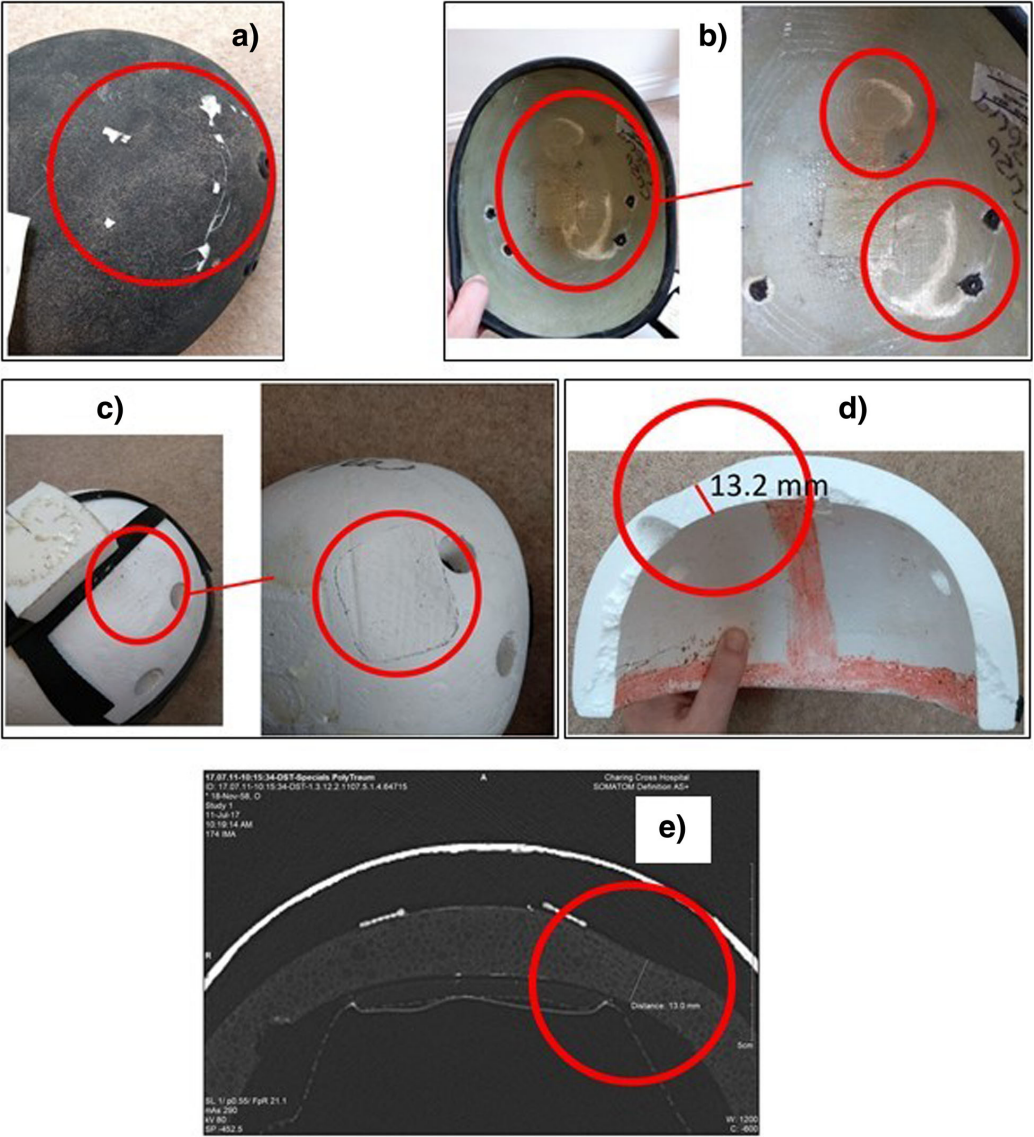
Example, identifying helmet damage. **a** Visible cracks can be seen on the outer surface of the fibreglass shell. One area of damage can be seen. **b** Visible delamination of the fibreglass shell can be seen in two distinct locations. Top and bottom of photo. The damaged area at the bottom of the photo corresponds to the external damage. **c** Visible crush of the EPS liner. **d** A section was cut through the centre of the damaged area and the liner thickness was measured at its thinnest point. It measured 13.2 mm. **e** Reviewing the CT images showed clear crushing of the EPS liner. At its thinnest point, the crushed region measured 13 mm in this case

All visual inspection was carried out before injury data was added in order to ensure that the inspection was effectively assessed blind with respect to head injury diagnoses. Helmets were inspected visually for any evidence of damage (Fig. 3) such as chipped paint, cracks/stress marks in the outer shell, tears in the fabric cover, if fitted, or overall deformation of the helmet. For composite shells, an acoustic coin tap test [18, 19] was used as a non-destructive method to detect variations in adhesive bonds. To ensure that visual inspection was a sufficient technique to evaluate helmet damage, 14 representative helmets were chosen and CT scanned (7 showing obvious external damage and 7 showing no obvious damage). CT images showed clear helmet liner crush and shell damage (Fig. 3e). Surprisingly, however, visual inspection revealed more detail in some cases such as impact stress lines on the inside of the helmet shell. Therefore, it was decided that visual inspection was the most accurate, expeditious and cost-effective method to identify and quantify the extent of helmet damage.

### Helmet disassembly

Disassembly of helmets was required to inspect any internal damage. In most cases, this was achieved by steaming the outer shell of helmets. This process melted the glue between the energy absorbing liner and the shell, allowing both parts to be separated without damaging either component. Some helmets were simply disassembled by manually pulling the energy absorbing liner from the shell while others required a cut to be made to the outer shell from front to rear using a cutting disc.

### Identifying impact locations

Impact locations were identified visually, either via delamination, cracking or stress marks on the composite and plastic shells or via residual crush of the energy absorbing liner. The identified area of damage was outlined, and the impact location was taken to be at the approximate centre of the damaged area (Fig. 3a–c). It was presumed that observed impact damage was the result of a single impact at that location.

### Measuring residual crush

Maximum residual crush of the energy-absorbing liner was measured at the centre of impact. In most cases, residual crush could be determined by comparing the crushed side of a liner to the corresponding undamaged side (Fig. 3d). For 12 common models, a new exemplar helmet was purchased for reference. In cases where the impact occurred on or near the centre line, the reference was taken from the nearest undamaged material.

### Comparing Snell- vs. non-Snell-certified helmets

To test if there was any difference between Snell and non-Snell helmets in terms of reported head injury, a chi-squared test was carried out for Snell- versus non-Snell-certified helmets, damaged and undamaged for all head injuries.

### Patient involvement

Following an accident, riders or their doctors filled in an accident report form which detailed their injuries and gave a description of the accident. Those participating in the helmet return schemes gave informed consent for their data to be used in this and subsequent studies. There was no further involvement from any rider.

## Results

### Impact damage

Helmets collected ranged in size from 53 to 62 cm internal circumference with a mean of 56.8 cm. The equestrians who used the helmets were 27% male and 73% female, aged between 11 and 67 years old with a median age of 27 years old. The equestrian demographic is largely representative of the population of equestrians [20]. Nineteen percent of equestrians were professional, and 81% were amateur.

Of the 216 helmets analysed in this study, 129 (60%) were found to have damage consistent with an impact. Eighty-seven (40%) helmets showed no evidence of damage whatsoever from an impact. Forty-eight (50%) of the damaged helmets had some external visible damage. Of the 129 damaged helmets, a single impact was found on 112 (87%) helmets, two distinct impacts were found on 14 (11%) helmets and three distinct impacts were found on 3 (2%) helmets. Therefore, a total of 149 impact sites were found in all. It is not known if multiple-impact damage to the same helmet was as a result of more than one accident.

### Injuries and associated damage

Of the 216 helmets analysed, 189 (88%) riders reported whether they sustained an injury or not. Of the 189 cases, 139 (70%) had an associated head injury. Seventy-five (54%) of the head injury cases had an associated damaged helmet, and 64 (46%) helmets showed no evidence of damage. Injuries reported for undamaged helmets were 1 subdural haematoma, 1 cerebral edema and 62 concussions. Of the 139 reported head injuries, 126 (91%) were concussions, 6 (4%) skull fractures, 1 (0.7%) subdural hematoma, 1 (0.7%) cerebral edema and 5 (3.6%) diffuse axonal injury (DAI). Table 2 summarises the injuries and associated helmet damage. Reported symptoms included temporary loss of vision, loss of hearing, both long- and short-term memory loss and reduced mental capacity. One rider died from their head injuries. Carrying out a chi-squared test for Snell- versus non-Snell-certified helmets, damaged and undamaged for all head injuries yielded *p* < 0.001.

**Table 2:** Summary of reported head injury and associated helmet damage (percentage of total number in brackets). Note: Most helmets were certified to two or more standards

	Total no.	Equestrian type		Helmet certification			
		Professional	Amateur	Snell	PAS015	EN1384	ASTM
No. of helmets	216	40 (19%)	176 (81%)	43 (20%)	169 (78%)	133 (62%)	101 (47%)
No. of injury reports	189	29 (15%)	160 (85%)	43 (23%)	147 (78%)	106 (56%)	87 (46%)
No. of head injuries	139	10 (7%)	129 (93%)	34 (24%)	102 (73%)	70 (50%)	65 (47%)
No. of head injury cases with damaged helmet	75	10 (13%)	65 (87%)	9 (12%)	64 (85%)	43 (57%)	42 (56%)
No. of head injury cases with undamaged helmet	64	0 (0%)	64 (100%)	25 (39%)	38 (59%)	27 (42%)	23 (36%)
Concussion	126	10 (8%)	116 (92%)	34 (27%)	93 (74%)	61 (48%)	56 (44%)
Skull fracture	6	0 (0%)	6 (100%)	0 (0%)	6 (100%)	6 (100%)	6 (100%)
Haematoma	1	0 (0%)	1 (100%)	0 (0%)	0 (0%)	1 (100%)	1 (100%)
cerebral edema	1	0 (0%)	1 (100%)	0 (0%)	1 (100%)	1 (100%)	1 (100%)
DAI	5	0 (0%)	5 (100%)	0 (0%)	5 (100%)	5 (100%)	5 (100%)

## Discussion

### Helmet damage and injury

Equestrian helmets work by attenuating impact energy. In general, this is achieved by the destruction (crushing) of the main functional component (the energy absorbing liner) and, to some degree, the outer shell. The impact must be of a minimum severity before the helmet begins to permanently crush. For less severe impacts, some energy may be attenuated elastically, after which the helmet remains undamaged. Only 60% of the helmets analysed in this study showed evidence of impact damage (either shell damage, liner damage or both), which shows that a significant proportion did not reach their threshold for permanent damage. One may argue that these are simply low-severity cases and that damage should not be expected. However, of the 139 head injury cases, 46% had undamaged helmets associated with them. These impacts were sufficiently severe to cause concussion and, in two of these cases, subdural haematoma and cerebral edema were also reported.

There are a number of reasons why this may be the case. Helmets are designed for impact against rigid surfaces. Real-world surfaces, however, tend to be much softer as horse riding predominantly occurs on turf or sand. Helmets are much stiffer than many of these surfaces, and therefore, the surface will deform before the helmet does during an impact. This difference in relative stiffnesses may explain the absence of helmet damage in some cases but it does not explain the occurrence of head injury.

Head injuries may result from rotational acceleration. Rather than a vertical drop onto a flat surface (causing linear or translational acceleration of the head), the head impacts the ground obliquely, inducing rotation of the head (causing rotational acceleration). Studies have shown that the brain is more sensitive to rotational acceleration than translational motion and that oblique impacts can cause concussion, diffuse axonal injury and subdural hematoma [21–23]. Additionally, oblique impacts tend to cause less damage to a helmet than translational impacts as some of the energy is used to rotate rather than compress the helmet.

It is possible that the injury cases with undamaged helmets resulted from an oblique impact against a compliant surface; however, more data is needed to prove this. Such kinematic conditions are addressed by current equestrian certification tests.

The data in this paper should not be misinterpreted to infer that helmets have no influence on the severity of head injury sustained.

### Helmet standards and injury

Changes in helmet certification tests over the past three decades have primarily been centred on increasing impact velocities and reducing the impact threshold levels that must be attained by a helmet. Although these test criteria have become more stringent, ensuring that the current generation of helmets can absorb more energy, the tests have remained fundamentally unchanged. They were originally designed to reduce the risk of death by preventing skull fracture and the pass/fail threshold was set with this purpose in mind [24–26]. Only recently have international standards bodies begun to develop new test methods in an effort to reduce incidences of concussion [27]. These new test methods introduce an oblique impact test in which rotational acceleration and velocity are analysed as well as translational acceleration. Additionally, it has been proposed that data from this new test be used as an input to finite element brain models [28–30] to better understand which helmets are most effective at protecting against oblique impacts. The need for these new test methods is shown very clearly by this study, particularly as 91% of all reported injuries were concussions. Four percent of all reported injuries were skull fractures, which suggests that helmet designs could be improved further to provide even greater protection against such injuries. Indeed, in many cases, helmets were quite badly damaged and showed significant crushing of the energy-absorbing liner, suggesting that they were to have likely prevented more serious head injury or death.

Certification tests tend to focus on these more extreme cases. Helmets tested to Snell standards (Table 1) are particularly stringent. They have the highest test impact velocity and are also dropped onto all three anvil types (Fig. 2). Those helmets have a stiffer shell and a higher density energy-absorbing liner than helmets tested to other standards. Twenty percent of helmets in this study were certified to a Snell standard, and they make up a disproportionate percentage (39%) of undamaged helmets that had an associated head injury. Carrying out a chi-squared test for Snell versus non-Snell certified helmets, damaged and undamaged for all head injuries, yields *p* < 0.001. This suggests that stiffer helmets may be less effective for lower severity impacts as helmet stiffness could be considered the greatest difference between Snell- and non-Snell-certified helmets and as Snell-certified helmets are more likely to remain undamaged even if the wearer sustained a head injury. Therefore, in real-world accidents, more protection may be provided for a rider’s brain by a means of a more compliant helmet, which would dissipate more impact energy rather than it being transmitted through the helmet to the rider’s head and brain.

### Clinical implications of results

The data suggest that equestrian helmets may not be effective at preventing concussion, and so future helmet designs should consider the long-term consequences of such injuries. Additionally, physiological differences between young children, adults and elderly riders could usefully be explored as there is little evidence in the literature about how these differences relate to head injury, even for cycle helmets (a much greater population).

The effect of multiple-impact injuries will be influenced by the time span over which a person has been riding throughout their life. The effects of cumulative head injuries in sports including rugby, boxing, soccer and football is increasingly recognised as a potential risk factor for long-term cognitive decline and even dementia. The repetitive nature of head injuries in equestrian sports [31] must come with a similar risk, albeit at possibly different risk levels.

### Limitations

This was a retrospective study. Helmets collected for this study came from several sources as outlined in the “Sample” section and the criteria by which helmets were submitted to this study differed between sources. It is possible that some helmets were only submitted by the rider when they sustained an injury or if their helmet had obvious damage. This would have the effect of skewing the data towards a higher occurrence of head injury than may actually be the case when horse riders sustain an impact to the head from a fall.

It is also possible that there was a low response rate and helmet wearers simply did not return their helmets following an accident which may have resulted in sampling bias; however, this is impossible to determine with the data available. Possible sampling bias may also have resulted in an over or underestimation of the effect of stiffer helmets on head injury frequency.

Following an accident, the helmet was sent to the research team by third parties. It is not possible to determine if those helmets were further damaged in transit. Additionally, it is not possible to determine if the helmet was damaged before the accident took place or if multiple impacts occurred at the same impact site.

Despite the limitations of this study, the data presented is the only data collected on this subject. The helmet collection schemes described were and are the only source of real-world equestrian accident helmets. This is the first time ever that any equestrian helmets have been collected and analysed in any detail, and it involved the active cooperation of many partners. The sample used was the best available at the time. Subsequent work is intended to carry out a long-term prospective study that will address the above mentioned limitations.

### Future work

New helmet standards must be informed by detailed accident reconstruction, clinical outcome data and the needs of helmet users. A discussion between engineers, clinicians, riders and equestrian regulatory authorities regarding acceptable risk, new helmet designs and long-term health consequences following head injury is needed. Data collected in this study will be analysed further in conjunction with experimental results in order to provide primary evidence as a basis for the next generation of equestrian helmet certification tests.

## Conclusions

Equestrian helmets may be effective at preventing skull fracture but the data presented in this study show high concussion incidence rates with current designs of helmets which may highlight an area where helmet standards and, in turn, helmet designs could be improved. The proportion of undamaged helmets with an associated head injury suggests that some helmets may be too stiff relative to the impact surface to reduce the risk of traumatic brain injury (TBI). It is also shown that helmets certified to the most stringent standard are overrepresented in this undamaged group. It may be possible to improve helmet designs and associated certification tests to reduce the risk of head injury in low-severity impacts. Future helmet certification tests must have an evidence basis and this should be informed by engineering and clinical data and have due regard for the needs of helmet users.

## Abbreviations

ABS: Acrylonitrile butadiene styrene
CT: Computed tomography
DAI: Diffuse axonal injury
EPP: Expanded polypropylene
EPS: Expanded polystyrene
mTBI: Mild traumatic brain injury
TBI: Traumatic brain injury
